# RNA Profiles of Tear Fluid Extracellular Vesicles in Patients with Dry Eye-Related Symptoms

**DOI:** 10.3390/ijms242015390

**Published:** 2023-10-20

**Authors:** Tanya Cross, Reidun Øvstebø, Berit Sletbakk Brusletto, Anne-Marie Siebke Trøseid, Ole Kristoffer Olstad, Trude Aspelin, Catherine Joan Jackson, Xiangjun Chen, Tor Paaske Utheim, Kari Bente Foss Haug

**Affiliations:** 1 The Regenerative Medicine Unit, Department of Medical Biochemistry, Oslo University Hospital, Ullevål, 0450 Oslo, Norway; tanyags@student.odont.uio.no (T.C.);; 2 Blood Cell Research Group, Department of Medical Biochemistry, Oslo University Hospital, Ullevål, 0450 Oslo, Norway; reidunovst@gmail.com (R.Ø.);; 3 Department of Ophthalmology, Sørlandet Hospital Arendal, 4838 Arendal, Norway; 4 The Norwegian Dry Eye Clinic, 0369 Oslo, Norway; 5 Department of Ophthalmology, Oslo University Hospital, 0450 Oslo, Norway; 6 Department of Ophthalmology, Vestfold Hospital Trust, 3103 Tønsberg, Norway

**Keywords:** dry eye disease, extracellular vesicles, RNA, tear film break-up time

## Abstract

Currently, diagnosing and stratifying dry eye disease (DED) require multiple tests, motivating interest in a single definitive test. The purpose of this study was to investigate the potential for using tear fluid extracellular vesicle (EV)-RNA in DED diagnostics. With a role in intercellular communication, nanosized EVs facilitate the protected transport of diverse bioactive molecules in biofluids, including tears. Schirmer strips were used to collect tears from 10 patients presenting with dry eye-related symptoms at the Norwegian Dry Eye Clinic. The samples comprised two groups, five from patients with a tear film break-up time (TBUT) of 2 s and five from patients with a TBUT of 10 s. Tear fluid EV-RNA was isolated using a Qiagen exoRNeasy Midi Kit, and the RNA was characterized using Affymetrix Clariom^TM^ D microarrays. The mean signal values of the two groups were compared using a one-way ANOVA. A total of 26,639 different RNA transcripts were identified, comprising both mRNA and ncRNA subtypes. Approximately 6% of transcripts showed statistically significant differential abundance between the two groups. The mRNA sodium channel modifier 1 (SCNM1) was detected at a level 3.8 times lower, and the immature microRNA-130b was detected at a level 1.5 times higher in the group with TBUT 2 s compared to the group with TBUT 10 s. This study demonstrates the potential for using tear fluid EV-RNA in DED diagnostics.

## 1. Introduction

Dry eye disease (DED) is a chronic condition characterized by both the loss of tear film homeostasis and ocular surface inflammation [1]. Symptoms of DED include eye irritation, pain, and visual impairment [2], which may have a considerable negative impact on both the quality of life and work productivity of those affected [3]. The prevalence of DED varies from 5 to 50% depending on the population studied [3], and both female sex and increasing age are consistently identified as important risk factors [3,4,5,6]. There are various aspects associated with aging that potentially contribute, including hormonal changes, an increase in the prevalence of autoimmune diseases, polypharmacy, and oxidative stress [5]. Additionally, while there is a higher prevalence among women, this appears to be more significant with age [3].

The tear film forms an interface between the cornea and the environment, and its integrity is essential for both the health of the eye and for visual acuity [7]. Lacrimal glands and epithelial goblet cells contribute to the tear film, and the normal function of these secretory elements is important for tear film homeostasis [7]. Multiple etiologies are implicated in DED, and several factors may contribute concurrently [8]. Traditionally, DED has been divided into two main categories: aqueous-deficient dry eye (ADDE), which implicates a reduction in the aqueous layer primarily due to lacrimal gland dysfunction, and evaporative dry eye (EDE), which is due to a disruption in the integrity of the tear film associated with meibomian gland dysfunction (MGD). Essentially, the impairment of any of the secretory elements and/or their regulation has the potential to interfere with tear film homeostasis and initiate DED. 

As DED encompasses diverse etiologies, there is a significant degree of heterogeneity between subjects; thus, the diagnosis and stratification of patients can be challenging. There is presently no single gold standard symptom or test [3,9]. Instead, a combination of diagnostic tests is often required. These include questionnaires, the measurement of tear fluid production and stability, the assessment of ocular surface inflammation, and the evaluation of meibomian gland function [9]. However, the clinical implementation and interpretation of these tests vary, and there are currently no international guidelines for a definitive DED diagnosis [10]. 

Although the pathophysiology of DED is incompletely understood, inflammation is considered integral to both the development and propagation of the condition [11]. A disruption in tear fluid homeostasis results in tear hyperosmolarity and ocular surface inflammation. The release of pro-inflammatory cytokines, such as interleukin-1ß, TNF-α, interleukin-6, chemokines, and matrix metalloproteinases [8], in turn, disrupts tear fluid homeostasis and osmolarity and consequently reinforces the inflammatory process. A vicious cycle is thereby established [11,12,13]. An interruption in homeostasis further affects the stability of the tear film, which may be assessed clinically by measuring the tear film break-up time (TBUT). 

**Figure 1:**
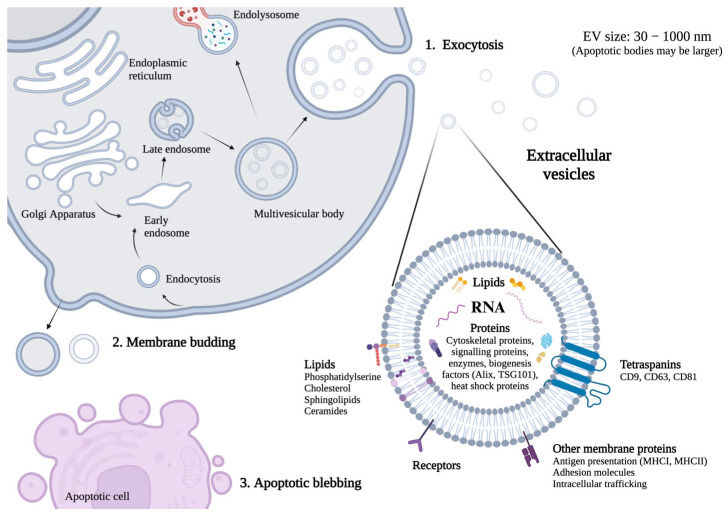
Extracellular vesicle (EV) biogenesis and cargo. There are three principal biogenetic pathways: (1) through endosomal multivesicular body formation and exocytosis, (2) via membrane budding, and (3) by apoptotic blebbing. EV cargo is both carried within the EV and associated with the phospholipid membrane. Created with BioRender.com.

Tear fluid collection is both non-invasive and relatively easy. The study of tear fluid has the potential to expand our understanding of DED and unveil new diagnostic opportunities. To date, minimal research has focused on the extracellular vesicle (EV) content of tear fluid. EVs are a heterogeneous group of nano-sized particles with a phospholipid membrane enclosing a cargo of nucleic acids, proteins, and lipids (Figure 1). They are produced by cells and released into the extracellular space. They may be categorized into three main groups based on their size and method of biogenesis: exosomes, microvesicles, and apoptotic bodies. Exosomes refer to small EVs ranging in size from ~30 to 200 nm. These are formed and released through the endosomal-multivesicular body (MVB) pathway and are released when the MVB fuses with the cell’s plasma membrane [14]. Microvesicles refer to EVs that are produced by direct budding of the plasma membrane [14,15], and these range in size from ~100 to 1000 nm. A third group, apoptotic bodies, are formed during programmed cell death and have a size distribution between 0.5 and 2 μm [16]. However, there is presently no definitive method to categorize EVs based on their biogenetic pathway, and as such, the International Society for Extracellular Vesicles (ISEV) proposes the use of the generic term “extracellular vesicle”, abbreviated “EV” [17]. 

EVs are present in all biological fluids and function in intercellular communication [16]. They may act locally (paracrine or endocrine) or at a distant site (exocrine function), and communication with the recipient cell may either be by ligand-receptor mediated signaling (no internalization), by direct fusion with the cellular membrane and transfer of cargo, or by endocytosis [18,19]. They have additionally been associated with extracellular matrix remodeling [16,20]. Their composition reflects the cell of origin, the mode of biogenesis [16], and the pathophysiologic state of the cell [18]. Understanding the content of EVs may provide useful information to distinguish between health and disease states. The RNA cargo is of particular interest due to the role RNA plays in gene regulation [21]. EVs contain an assortment of both protein-coding (mRNA) and non-coding (ncRNA) RNA subtypes [22], which are thereby afforded protection from nucleases. The transfer of RNA by EVs to recipient cells has been shown to generate a cellular response [23]. 

The primary aim of this study was, therefore, to characterize the RNA content of EVs isolated from the tear fluid of patients with symptoms of DED using microarray technology. Additionally, the secondary aim was to identify any potential differences in the abundance of specific EV-RNA transcripts between two groups of patients with dry eye-related symptoms, one with extremely low tear film stability (TBUT 2 s) and one with near normal tear film stability (TBUT 10 s). 

## 2. Results

### 2.1. EV Characterization

#### 2.1.1. Nanoparticle Tracking Analysis

The concentration of EVs, as determined by nanoparticle tracking analysis (NTA), was ~10^8^ particles/mL. The size distribution was between ~100 and 430 nm, and aggregates were seen. 

#### 2.1.2. Transmission Electron Microscopy

**Figure 2:**
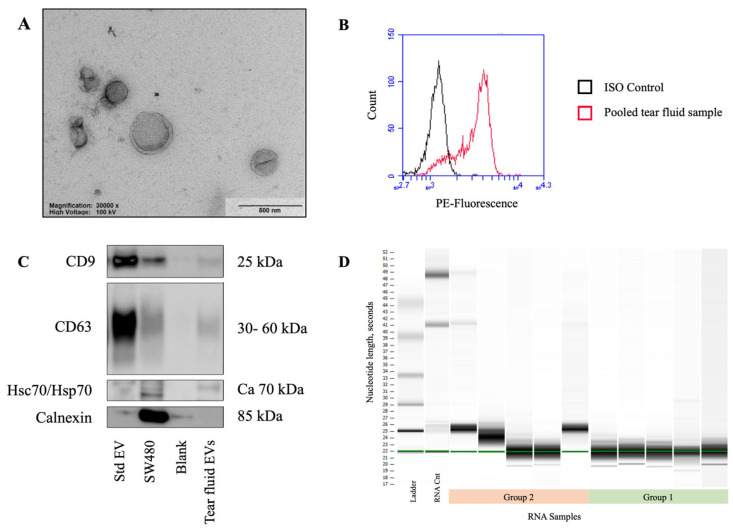
The characterization of extracellular vesicles (EVs) in pooled tear fluid samples. (**A**) A transmission electron microscopy (TEM) image of EVs. Magnification 30,000×. Image courtesy of Espen Stang, Ph.D. (**B**) Flow cytometry detection of EVs with PE-conjugated anti-CD9 antibodies. ISO = isotype control. Fluorescence values on the *x*-axis are scaled logarithmically. (**C**) Western blotting of EV proteins. CD9 and CD63 = EV membrane tetraspanins; Hsc70/Hsp70 = heat shock protein 70; calnexin = a negative control protein marker; Std EV = a recombinant EV standard; SW480 = a positive control cell lysate; kDa = kilodaltons. (**D**) The characterization of EV-RNA in all samples by an Agilent Bioanalyzer. Ladder RNA peaks = 25, 200, 500, 1000, 2000, 4000 nucleotides (nt); RNA Cnt = total cellular RNA control; green marker = internal standard, 25 nt.

Transmission electron microscopy (TEM) allowed for the direct visualization of EVs, as evidenced by cup-shaped particle morphology and a size range between 100 and 350 nm. TEM provided confirmation of the presence of EVs in the pooled tear fluid sample (Figure 2A). 

#### 2.1.3. Flow Cytometry

Flow cytometry analysis, using anti-CD9-coated magnetic beads and phycoerythrin (PE)-coupled anti-CD9 detection antibodies, indicated an increased level of the membrane-associated protein marker, tetraspanin CD9, in a pooled tear fluid EV sample relative to the isotype control (Figure 2B). This corresponded to a ΔMFI (delta mean fluorescence intensity) of 3500.

#### 2.1.4. Western Blotting

Western blotting analysis verified the presence of EV marker proteins in a pooled tear fluid sample following EV isolation using a modified Qiagen exoRNeasy column approach (Figure 2C). The membrane-associated marker proteins CD9 and CD63, as well as the cytosolic-associated EV marker, heat shock protein 70 (Hsc70/Hsp70), were detected in both the tear fluid EV sample and the commercial standard EV control sample. Additionally, the EV negative control protein calnexin, an endoplasmic reticulum protein absent in EVs, was not detected in the EV samples but was seen in the cell line SW480 sample (spill-over is visible in the neighboring columns).

#### 2.1.5. EV-RNA Characterization

The EV-RNA from each tear fluid sample was quantified and quality-assessed using Qubit^®^ Fluorometer, Nanodrop™, and Agilent Bioanalyzer analysis. The fluorometric analysis showed that group 1 (patients with TBUT 2 s) had an average EV-RNA concentration of 2.3 ng/µL, with a range of 1.9 ng/µL to 3.0 ng/µL. For group 2 (TBUT 10 s), the average EV-RNA concentration was 1.9 ng/µL, with a range of 0.6 ng/µL to 2.7 ng/µL. Based on the Agilent Bioanalyzer analysis, most of the RNA in all samples showed lengths of approximately 200 nucleotides or shorter (Figure 2D). 

### 2.2. Tear Fluid EV-RNA Analysis Using Microarrays 

#### 2.2.1. RNA Subtypes Identified

RNA was successfully detected in all tear fluid EV-RNA samples, both confirming the presence of RNA in tear fluid EVs and demonstrating the effective application of Affymetrix Clariom™ D microarrays in EV-RNA analysis.

Of the ~540,000 human RNA transcripts presented on the microarray chips, a total of 26,639 transcripts were detected (4.9%). The (mean) signal values ranged from 3 to 24,568, corresponding to the relative abundance of each transcript in the samples. 

**Figure 3:**
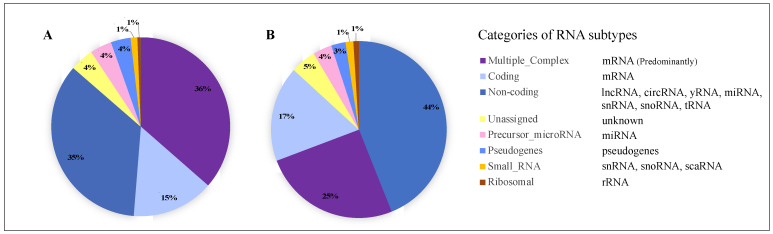
The categorization of RNA subtypes according to Affymetrix allocation. (**A**) All 26,639 RNA transcripts detected. (**B**) Transcripts with statistically significant differential abundance between group 1 (TBUT 2 s) and group 2 (TBUT 10 s), *p*-value < 0.05, n = 1598.

Most RNA subtypes so far defined were identified in the samples, including messenger RNA (mRNA), long non-coding RNA (lncRNA), microRNA (miRNA), circular RNA (circRNA), small nuclear RNA (snRNA), small nucleolar RNA (snoRNA), Y-RNA (yRNA), small Cajal body-associated RNA (scaRNA), ribosomal RNA (rRNA), and transfer RNA (tRNA). The RNA transcripts were categorized according to their RNA subtype, as defined by Affymetrix, and the comparative abundance of the different RNA subtypes was determined (Figure 3A). The category ‘Multiple_Complex’ incorporated principally mRNAs, as well as several ncRNAs. The category ‘non-coding’ predominantly comprised lncRNAs. Accordingly, it can be extrapolated that the most abundant RNA subtype detected in the tear fluid EVs was mRNA, and the most abundant ncRNA was lncRNA. 

#### 2.2.2. Comparing Patients with TBUT 2 s and TBUT 10 s

When group 1 (TBUT 2 s) was compared with group 2 (TBUT 10 s), 1598 transcripts were seen to have statistically significant differential abundance (*p* < 0.05). ncRNAs represented a slightly higher percentage of these transcripts compared to mRNA (Figure 3B). None of the transcripts had *q*-values < 0.05.

**Figure 4:**
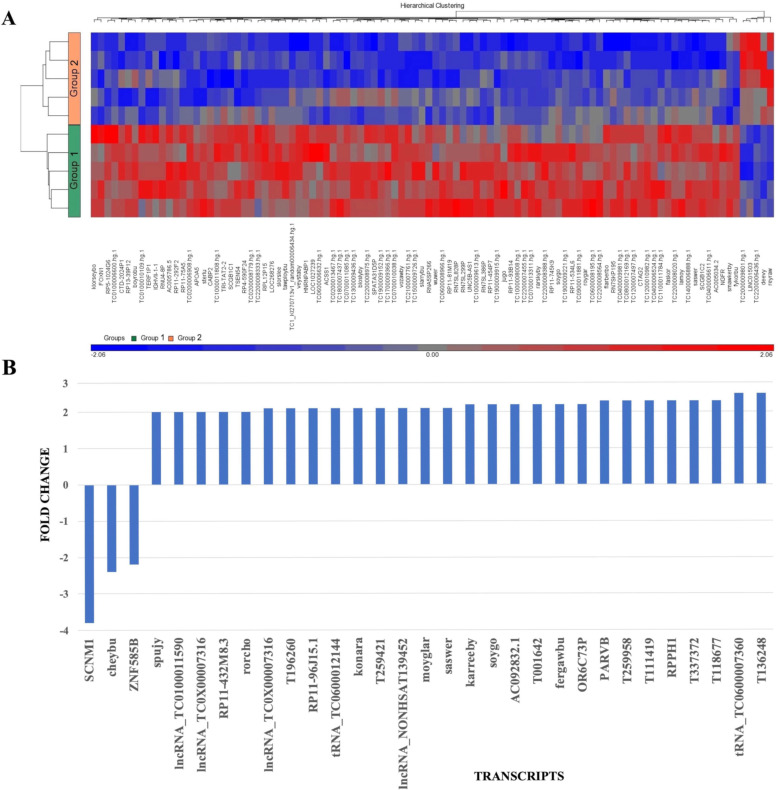

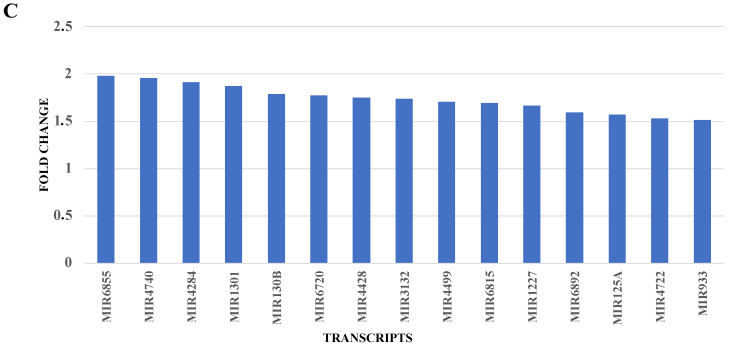
Comparing RNA transcript levels between group 1 (TBUT 2 s) and group 2 (TBUT 10 s). (**A**) A hierarchical cluster heatmap of the 100 transcripts with the most statistically significant difference in signal values between group 1 and group 2, *p*-value < 0.05. Red indicates a higher level of transcript relative to blue. (**B**) RNA transcripts with a statistically significant different level of abundance between group 1 and group 2, signal value ≥ 20, fold change ≥ 2. Positive fold change = higher levels of transcript in group 1 vs. group 2. Negative fold change = lower levels of transcript in group 1 vs. group 2. (**C**) Immature miRNAs with a statistically significant different level of abundance between group 1 and group 2, signal value ≥ 20, fold change ≥ 1.5.

A hierarchical cluster heatmap of the 100 transcripts with the most statistically significant difference in signal values between the two groups is presented in Figure 4A. Most of these transcripts showed higher levels in group 1 compared to group 2, and the majority of identifiable transcripts were mRNA. However, signal values were generally low. 

Transcripts with *p*-values < 0.05, signal values ≥ 20, and a fold change ≥ 2 were selected and presented graphically (Figure 4B). Of the 31 transcripts identified, 28 had higher levels in group 1. The protein coding gene Sodium Channel Modifier 1 (SCNM1) was present at a significantly lower level in group 1 relative to group 2, with a fold change of −3.8. 

Of the 1598 transcripts with statistically significant differential abundance between the groups, 34 were immature miRNAs. Of these, 15 had signal values ≥ 20 and a fold change ≥ 1.5 (Figure 4C).

#### 2.2.3. RNA Validation

RT-qPCR was used to validate two selected mRNA transcripts (Ribosomal Protein L9 (RPL9) and beta-2-microglobulin (B2M)) in three samples and one ncRNA transcript (yRNA1) in four samples. The results showed transcript levels consistent with the microarray signal values. 

## 3. Discussion

The Affymetrix microarray analysis of tear fluid EV-RNA performed in this study produced a large dataset of transcripts describing a diverse selection of RNA subtypes in all samples. A comparison of the RNA profiles between the two categories TBUT 2 s (group 1) and TBUT 10 s (group 2) identified several mRNA and miRNA transcripts with statistically significant differential abundance between the groups. While no clear RNA patterns or biomarker candidates were revealed (all *q*-values > 0.05), the study demonstrated the potential for using tear fluid EV-RNA in diagnostics, with further investigation recommended.

RNA is a large family of molecules composed of many different RNA subtypes, including protein-coding RNA (mRNA) and non-coding RNA (ncRNA). Together, the different RNA subtypes form a complex, intricate network of interactions controlling a multitude of cellular processes [21]. It has previously been recognized that the RNA carried by EVs differs substantially from total cellular RNA, in both subtype and quantity [18,24,25]. In this study, the most common RNA subtype detected in tear fluid EVs was mRNA, while lncRNAs made up the majority of the ncRNAs. Additionally, over 150 circRNAs were detected. The small ncRNAs, miRNA, snRNA, snoRNA, tRNA, scaRNA, and yRNA, were also detected at varying levels in all tear fluid EV-RNA samples. Here, it should be mentioned that the profile of small RNAs observed in EVs is influenced by both the EV and EV-RNA isolation methods utilized [26]. Finally, low levels of rRNA were measured in the tear fluid EV-RNA samples. This is consistent with the knowledge that rRNA is minimally represented in EV-RNA compared to cellular RNA [25,27], with a notable absence of the 18S and 28S ribosomal subunits [25].

There is general agreement that EV-RNA is involved in intercellular communication [18,28]. While originally surmised to be capsules for cellular waste disposal [15,29], EVs are now also considered entities that package bioactive molecules. The RNA cargo is of particular interest by virtue of its genetic messaging and modulatory capacity in recipient cells. The first RNA subtypes shown to be transferred by EVs were mRNA and miRNA [30], and they have also been shown to be functional after uptake in target cells [23,30]. This study found an abundance of mRNA and a substantial level of miRNA in the tear fluid EVs. Interestingly, mRNA is now known to not only function as a template for translation to a protein but has also been demonstrated to have regulatory roles [31]. Additionally, this regulatory capacity may be attributed to mRNA fragments rather than whole transcripts. The miRNA detected by the Clariom™ D microarrays is, however, the immature form. Therefore, there is uncertainty regarding the function of these miRNAs once transferred to recipient cells. 

There remain many facets relating to EV-RNA intercellular communication that still need to be resolved. Little is known about the selectivity of RNA into EVs or the fate of the RNA after it has been delivered into the target cell [32]. Adding to this complexity is the knowledge that RNA transcripts in EVs are short in length, with sizes ranging from 20 to 200 nucleotides [32]. The tear fluid EV-RNA in this study was also shown to be in accordance with this, as measured by the Bioanalyzer analysis. Given that the majority of RNAs detected were mRNA and lncRNA, which have average native lengths well over 200 nm, it is likely that the detected transcripts within EVs were either fragmented or degenerated. This further complicates the understanding of the functionality of the RNA contained in EVs. Are the transcripts purposely packaged in fragmented form, or has the RNA deteriorated post-collection? What, if any, are the roles of the altered transcripts? It has, for instance, been hypothesized that fragmented forms of lncRNA, snRNA, and tRNA may function like miRNA [21]. Further, lncRNA, circRNA, and mRNA may contain elements that compete for miRNA binding and thereby act as miRNA sponges [21]. This proposes an expanded interplay between the various RNA subtypes, of both whole and fragmented form, and highlights the complexity of a cell’s regulatory network.

While all subjects included in the current study showed clinical signs of DED, their clinical presentation varied considerably. Unfortunately, present diagnostic tests have a relatively low reproducibility [33] and cannot categorize the severity of DED accurately and simply. This is in part due to the multifaceted nature of DED and partly due to the weak correlation between subjective symptoms and diagnostics [34,35], with each diagnostic measurement providing instead a discrete detail about the state of the ocular surface. TBUT is one of the most frequently used clinical methods to evaluate dry eye disease, with values <10 s generally considered an indication of DED [9]. This is normally performed following the installation of fluorescein, and it describes the time from the last complete blink until the first break in the tear film [36]. A value of 10 s is considered marginal. In this study, the EV-RNA from patients with two different rates of TBUT were compared, where group 1 had abnormal tear film break-up times (TBUT 2 s), and group 2 had close-to-normal tear film break-up times (TBUT 10 s). These values were then chosen to represent the upper and lower limits of tear film stability. 

**Figure 5:**
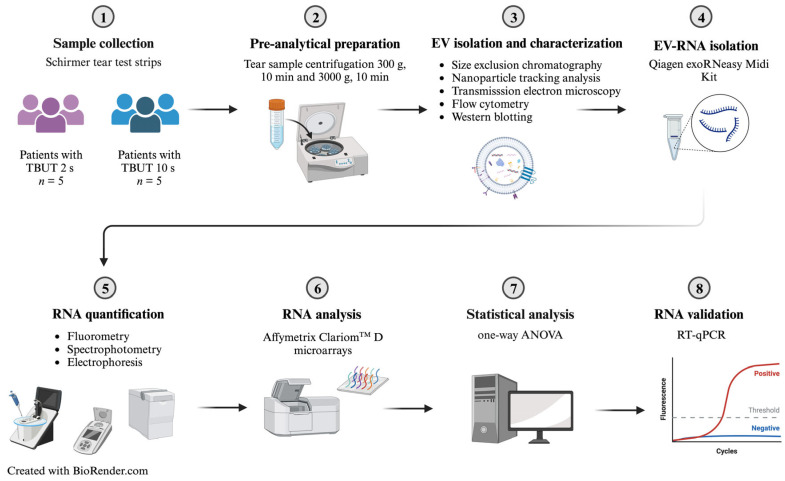
Study workflow. TBUT = Tear film break-up time. Adapted from Figure 6 in Ref [57].

Although no transcripts were identified with clear RNA biomarker potential for tear film instability, 6% of the transcripts were seen to have differential abundance between the EVs of group 1 and group 2. The comparison of these profiles (Figure 5) revealed that, for most of these transcripts, there is little or no previous reference to them in the literature or information available in databases, highlighting the novelty of their detection. However, two transcripts are worth a remark based on their prior recognition. Firstly, the protein-coding gene Sodium Channel Modifier 1 (SCNM1) showed close to a 3.8 times lower level in group 1. SCNM1 codes for a component of the minor spliceosome [37,38,39], a protein believed to be involved in stabilizing the complex [38]. Whilst the functionality of the EV-RNA cargo was not the focus of this study, the involvement of this gene in DED warrants further investigation. Similarly, the immature mir-130b was detected at a level 1.5 times higher in group 1 compared with group 2. Other research has shown that the mature form of this miRNA is involved in various disease processes. Pucker et al. identified the upregulation of miR-130b-5p in tear fluid EVs of dry eye patients compared to healthy controls, conjecturing that miR-130b-5p may be associated with inflammation [40]. Several other studies have also predicted the involvement of miR-130b in both inflammation [41] and cancer [42,43]. The current findings thereby support the potential role of mir-130b in the pathogenesis of DED. 

Tear fluid is composed of secretions from lacrimal glands (main and accessory), meibomian glands, conjunctival goblet cells, and epithelial cells of the ocular surface. As such, the cellular origins of tear fluid EVs are presumably quite diverse. To date, only a minor number of publications have focused on EVs in tear fluid [40,44,45,46,47,48,49,50,51,52,53,54]. Each of these studies employed different methods of EV isolation and analysis and focused on various disease scenarios. Only four of these studies were primarily focused on EVs isolated from the tear fluid of dry eye patients [40,45,46,51], and only one (Pucker et al.) focused on the RNA content of tear fluid EVs, while the other three analyzed the proteome of tear fluid EVs. Pucker et al. used RNA sequencing rather than microarray technology and focused only on the miRNA content of EVs [40]. To the best of our knowledge, the work presented here is the first study reporting the microarray profiling of both coding and non-coding RNA in tear fluid EVs. As such, there is very little published information available for the comparison of results or for further gene ontology analysis. Microarray analysis was chosen as the method to analyze the RNA because of the relative ease it allows for the quantitative comparison of large numbers of known transcripts between two groups, as well as the minimal requirement for post-analytical bioinformatic processing. Additionally, the microarrays used represent human-only transcripts, such that the EV-RNA of microbial origin are automatically excluded. 

This current study employed three novel methods of biomolecular analysis for studying tear fluid EVs and tear fluid EV-RNA. Firstly, a Qiagen exoRNeasy EV-RNA isolation kit was used to isolate EV-RNA from tear fluid. Secondly, Affymetrix microarrays were used to analyze the EV-RNA. Additionally, a Qiagen exoRNeasy affinity column was used with RIPA lysis buffer, in a modified approach, to isolate tear fluid EV proteins for use in Western blotting. Ideally, the EVs used for characterization should be isolated by the same method used for EV-RNA analysis; however, the Qiagen exoRNeasy kit combines both EV and RNA isolation. This did not enable the extraction of EVs for characterization. SEC was originally used to isolate EVs from the tear fluid; however, the yield was too low for use in characterization, even with pooled samples. This was evident based on the results from the NTA and initial pilot projects for flow cytometry and Western blotting. Therefore, the characterization of EVs was performed on pooled whole tear fluid samples (TEM and flow cytometry) and together with a modified Qiagen exoRNeasy column approach for Western blotting. Of further note, the particle concentration following SEC EV isolation as measured by NTA was below the manufacturer’s recommended range for result reliability (1–9 × 10^8^). As aggregates were seen, increasing the vortex time prior to NTA may be of benefit for future studies. 

The successful isolation and characterization of RNA from all the tear fluid EV samples opens new opportunities. However, a greater number of subjects would be encouraged for future comparative studies. The heterogeneity of the subjects’ ages, sex, and other diagnostic information reflects the heterogeneity of DED pathogenesis, and as such, including a larger number of subjects may help identify commonality in expression. The procurement of tear fluid samples from healthy controls would also provide a better basis for future comparison.

There are several methodological considerations worth highlighting. While tear fluid collection using Schirmer strips is a relatively standardized and convenient method, it does have the potential to induce reflex tear production and cause irritation of the conjunctiva, thereby affecting sample composition [55]. Additionally, collection volumes are generally low and extraction from the strips is imperfect. The use of capillary tubes for tear fluid collection has the potential to remedy these issues; although, this is a technique that requires more time and a high degree of competency. In addition, it is important to remember that EVs are not the only source of extracellular RNA in biological fluids. RNA is also found bound to other carrier molecules and lipoproteins [56]. These RNAs may also potentially contribute to the overall network of intercellular communication [32]. Furthermore, while Qiagen exoRNeasy EV-RNA kits are designed to capture only EV-RNA cargo, the inadvertent inclusion of other extracellular RNA should not be overlooked. The addition of RNase to the EV-eluate prior to RNA isolation should therefore be considered for future studies. In relation to the EV-RNA analysis, it is worth noting that Affymetrix Clariom™ D microarrays are designed for use with whole-cell RNA; hence, the repertoire of transcripts is not EV-specific. 

This study demonstrated the feasibility of analyzing the RNA content of tear fluid EVs using microarrays. It will be interesting to see if follow-up studies with larger group sizes can identify transcripts that may be used in DED diagnostics. 

## 4. Materials and Methods

The study workflow is presented in Figure 5.

### 4.1. Study Participants

Tear fluid samples were collected from the eyes of 10 first-time visitors at the Norwegian Dry Eye Clinic, all presenting with dry eye-related symptoms. Tear fluid from one eye per patient was used in this study. Informed consent was obtained from the patients, and the study was approved by the Regional Committee for Medical and Health Research Ethics. All patients completed a questionnaire (OSDI) and underwent a battery of diagnostic tests, including measurement of tear fluid production (Schirmer 1 test without anaesthesia), fluorescein tear film break-up time (TBUT), and assessment of ocular surface staining (OSS). Two groups of tear fluid samples were then selected according to the subjects’ TBUT; samples from patients with a TBUT of 2 s (considered extremely reduced tear film stability) were placed in group 1, and samples from patients with a TBUT of 10 s (considered close to normal tear film stability) were placed in group 2. An overview of the clinical data from each subject is presented in Table 1.

### 4.2. Tear Fluid Sample Collection

Schirmer tear test strips (HAAG-STREIT, Essex, UK) were used for tear fluid collection, for a period of five minutes. The strips were then placed in individual 1.5 mL collection tubes containing 500 µL phosphate-buffered saline (PBS). The samples were then frozen at −80 °C until use.

### 4.3. Tear Fluid Sample Preparation: Differential Centrifugation

Samples were thawed and individually centrifuged in a two-step differential centrifugation procedure. The samples were first centrifuged at 300× *g* for 10 min, RT, with the Schirmer tear test strips still in place. The strips were then removed, and the samples were again centrifuged at 3000× *g* for 10 min, RT, to remove cellular waste and debris. The supernatant was transferred to new tubes. A portion of each sample was directly used for EV-RNA isolation, and the remaining portion was frozen at −80 °C until further use in EV characterization. Thawed samples were pooled prior to EV characterization. 

### 4.4. EV Isolation and Characterization: Nanoparticle Tracking Analysis (NTA)

EVs were isolated from a pooled tear fluid sample using size-exclusion chromatography (SEC) (qEV 70 nm size-exclusion chromatography column, iZON Science, Oxford, UK). Fractions 7–10 were combined, and the size distribution and concentration of the EVs were determined by nanoparticle tracking analysis (NTA), using a NanoSight NS500 (Malvern Instruments Ltd., Amesbury, UK). The sample was diluted 1:1 with filtered PBS (0.02 μm filter, Whatman™ Anotop™ 25, GE Healthcare Life Science, Buckinghamshire, UK), vortexed, and then analyzed in triplicate. The camera level was set to 13, and the detection threshold was set to 3. Videos 60 s in length of a continuous flow of sample were recorded. Data analysis was performed using NTA 3.1 software. 

### 4.5. EV Characterization: Transmission Electron Microscopy

Transmission electron microscopy (TEM) was performed on a pooled tear fluid sample. Formvar carbon-coated copper grids were placed on top of 5 µL drops and followingly incubated for 5 min at RT, before being washed 3 times with distilled H_2_O. The grids were then incubated with 2% methylcellulose containing 0.3% uranyl acetate on ice for 10 min. The grids were then air-dried before being viewed with a Tecnai G2 Spirit transmission electron microscope (FEI, Hillsboro, OR, USA). A Morada digital camera and RADIUS imaging software (http://emsisasia.com/radius/) were used to create images, which were further processed using Adobe Photoshop. 

### 4.6. EV Characterization: Flow Cytometry Analysis 

Flow cytometry was performed on a pooled tear fluid sample. Aliquots of 20 μL Dynabeads^®^ (Exosome-Human CD9 Flow Detection Reagent, Invitrogen, Thermo Fisher Scientific, Oslo, Norway, Cat no 10620D) were added to 100 μL of the tear fluid sample and to each of three controls (negative control: assay buffer; positive control: SW−480 cell line EVs; ISO-control: tear fluid). Overnight incubation was performed in a HulaMixer Sample Mixer at 2–8 °C. Following wash steps, 150 μL of assay buffer was used to redistribute the beads, and 100 μL from each sample was then combined with 25 μL of PE-conjugated detection antibodies (CD9-PE, Cat no 555372, BD Biosciences, Oslo, Norway) or 25 μL of isotype-matched control (IgG1-PE, Cat no 559320, BD Biosciences, Oslo, Norway). An orbital shaker (IKA-WERKE GMBH & CO, Staufen, Germany) was then used for 45 min at 1000 rpm. Following two wash steps, a final 100 μL of assay buffer was added to each tube. All samples and controls were then placed on ice, ready for flow cytometry analysis. A BD Accuri™ C6 Cytometer (BD Biosciences, Oslo, Norway) was used for flow cytometry EV detection. 

### 4.7. EV Isolation and Characterization: Western Blotting of EV-Proteins Isolated from Tear Fluid 

Western blotting was performed on a pooled tear fluid EV-isolated sample. EV isolation was conducted using a modified Qiagen exoRNeasy EV isolation method (see Section 4.8). Briefly, 150 µL of pooled tear fluid was used in the first spin column steps of the exoRNeasy protocol. QIAzol, a lysis reagent, was replaced with 100 µL of a 1:5 ratio mix of protease inhibitor (Merck Life Science, Oslo, Norway Cat no 04693159001) and RIPA buffer (Thermo Fisher Scientific, Oslo, Norway, Cat no 89900). The sample was then sonicated for 20 s and incubated on ice for 15 min before five minutes of centrifugation at 5000× *g*. The lysate and controls, SW480 cell lysate and recombinant Exosome standard (Merck Life Science, Oslo, Norway, Cat no SAE0193), were heated to 70 °C for 10 min and then loaded on a 4–12% Bis-Tris gel (Bolt, Thermo Fisher Scientific, Oslo, Norway, Cat no NW04122BOX). CD9 and CD63 were examined under non-reducing conditions and Hsc70/Hsp and calnexin under reducing conditions. Proteins were blotted onto a PVDF membrane (Thermo Fisher Scientific, Oslo, Norway, Cat no LC2002), which was then blocked with casein before overnight incubation with primary antibodies. The antibodies used were anti-CD9, 1:750 (Invitrogen, Thermo Fisher Scientific, Oslo, Norway, Cat no 10626D), anti-CD63 1:600 (Invitrogen, Thermo Fisher Scientific, Oslo, Norway, Cat no 10628D), anti-Hsc70/Hsp 1:1000 (Enzo Life Science, AH Diagnostics AS, Oslo, Norway, Cat no ADI-SPA-820), and anti-Calnexin 1:1000 (Abcam, Cambridge, UK, Cat no ab22595). Secondary antibodies used were Rabbit TrueBlot and Mouse TrueBlot (Cat no 18-8816-33 and 18-8817-33, Rockland Immunochemicals, Pottstown, PA, USA). The blots were imaged using SuperSignal™ West Dura Extended Duration Substrate (Thermo Fisher Scientific, Oslo, Norway, Cat no 34075) with an Amersham™ Imager 600 (GE Healthcare Bio-Sciences AB, Uppsala, Sweden).

### 4.8. Tear Fluid EV-RNA Isolation Using a Qiagen exoRNeasy Midi Kit

EV-RNA was isolated from each tear fluid sample using a Qiagen exoRNeasy Midi Kit (Qiagen, Hilden, Germany), according to the manufacturer’s guidelines. A sample input volume of 300 µL was used. Following the QIAzol and chloroform steps, 200 µL of the aqueous phase, containing the RNA, was transferred to new tubes. A DNase step was included following the 100% ethanol-centrifugation step. RNAse-free water was used to recover the RNA, producing samples of approximately 12 µL. The RNA elutes were immediately placed on ice, prior to RNA quantification and characterization, and then frozen at −80 °C until further use.

### 4.9. Sample RNA Quantification and Purity Assessment Using a Qubit^®^ 2.0 Fluorometer and a Nanodrop™ One Spectrophotometer, Respectively 

Sample RNA concentrations were determined using a Qubit^®^ 2.0 Fluorometer (Life Technologies Corporation, CA, USA) combined with a Qubit^®^ microRNA Assay Kit (Invitrogen by Thermo Fisher Scientific, Hillsboro, OR, USA), according to the manufacturer’s guidelines. Sample volumes of 1 µL were used. The measurements obtained were then used to calculate the Affymetrix microarray sample input volumes. Additionally, contaminants (guanidine or phenol) were assessed using a Nanodrop™ One Spectrophotometer (Thermo Fisher Scientific, Oslo, Norway), using sample volumes of 1 µL.

### 4.10. Sample RNA Characterization Using an Agilent 2100 Bioanalyzer

Quality analysis was performed on 1 µL of each RNA sample using a Bioanalyzer (Agilent Technologies, Santa Clara, CA, USA) together with an Agilent RNA 6000 Pico Assay Kit (Agilent Technologies, Santa Clara, CA, USA) in accordance with the manufacturer’s guidebook. 

### 4.11. Extracellular Vesicle RNA Analysis Using Affymetrix Clariom^TM^ D Microarrays 

A GeneChip^TM^ WT Pico Reagent Kit (Cat no 902623) was used to prepare the RNA samples prior to their application to Clariom™ D microarray chips (both Thermo Fisher Scientific, Oslo, Norway). This was performed according to the manufacturer’s guidelines and using 2 ng of total EV-RNA per sample. A HeLa cell sample was used as a positive control with a total RNA input of 0.5 ng. Nuclease-free water was used as a negative control. Poly-A RNA was used as an exogenous positive internal control in all samples. A Veriti^®^ 96-Well Thermal Cycler (Applied Biosystems, 739256, Singapore) was used for the pre-IVT PCR amplification with 9 PCR cycles applied. The prepared samples of fragmented and labeled ss-cDNA were then injected into individual Clariom^TM^ D microarray chips, which were incubated for 17 h at 45 °C in an Affymetrix GeneChip 645 hybridization oven with a rotation of 60 rpm. Following the hybridization process, the microarray chips were immediately washed and stained in a GeneChip™ Fluidics Station 450 using a GeneChip™ Hybridization, Wash, and Stain Kit (Thermo Fisher Scientific, Oslo, Norway, Cat no 901241). The microarray chips were then scanned with a GeneChip^TM^ Scanner 3000 System and analyzed with GeneChip^TM^ Command Console^TM^ software, version 4.0. All Affymetrix instruments and software were from Affymetrix, Santa Clara, CA, USA.

### 4.12. RNA Validation Using RT-qPCR

Reverse transcriptase quantitative polymerase chain reaction (RT-qPCR) was used to validate the Affymetrix detection of selected RNA transcripts.

RT-qPCR was performed on three RNA samples to validate the presence of two mRNA transcripts (RPL9 and B2M). The transcripts were selected based on the relatively high signal values detected in the samples and the availability of the assays in the laboratory. Total cellular RNA was used as a positive control, and RNA-free water was used as a negative control. Reverse transcription (RT) was performed using a Veriti^®^ 96-Well Thermal Cycler and qPCR with a ViiA™ 7 Real-Time PCR Instrument (Applied Biosystems, 739256, Singapore). The reagents used were SuperScript™ Enzyme Mix (Cat no 11754-050) and VILO™ Reaction Mix (Cat no 11754-250) for RT, and Taqman™ Fast Advanced Master Mix (Cat no 4444557) together with Gene Expression Assays (RPL9 Taqman^®^ Gene Expression Assay, Hs01552541_g1 and B2M Taqman^®^ Gene Expression Assay, Hs99999907_m1) for qPCR, all provided by Thermo Fisher Scientific, Oslo, Norway.

Additionally, to substantiate the presence of an ncRNA transcript, the validation of yRNA1 was performed externally, at the Department of Microbiology, Oslo University Hospital, on four RNA samples.

### 4.13. Bioinformatic Processing

The fluorescent signal values of the RNA transcripts identified by the Affymetrix microarray analysis were recorded, and mean values were calculated for each group: TBUT 2 s (group 1) and TBUT 10 s (group 2). Partek^®^ Genomics Suite^®^ software (https://www.partek.com/) was used for the statistical analysis. A one-way ANOVA was applied to determine any statistically significant abundance between the groups, with *p*-values less than 0.05 considered to be indicative of statistical significance. Additionally, *q*-values and fold changes were determined. Furthermore, a principal component analysis (PCA) was performed on the data, and a cluster heatmap was produced using the 100 transcripts with the most statistically significant difference in signal values between the groups.

## 5. Conclusions

This study provides the novel characterization of the RNA content of tear fluid EVs from patients with dry eye-related symptoms, using microarray technology. Thousands of RNA transcripts, both mRNA and ncRNA, were successfully detected, supporting the potential for the use of tear fluid EV-RNA in DED diagnostics. Approximately half of the transcripts identified were coding RNAs, and approximately 6% of the transcripts showed a significant differential level of abundance between patient groups with TBUT 2 s and TBUT 10 s. While the comparison of RNA profiles revealed no conclusive results, the protein-coding gene SCNM1 and the immature mir-130b stood out as genes of interest. These findings may be useful in guiding and supplementing further research into tear fluid EV-RNA. Additionally, future knowledge concerning the roles of the different ncRNA subtypes may reveal information yet undisclosed.

## Figures and Tables

**Table 1:** Clinical data.

Group	Age	Sex	TBUT (s)	OSDI	Schirmer (mm)	OSS
1	59	F	2	33.3	9	0
1	66	F	2	47.9	20	0
1	22	M	2	37.5	6	1
1	20	M	2	39.6	14	2
1	75	F	2	45.5	35	3
2	47	M	10	8.3	5	2
2	51	F	10	20.8	30	1
2	39	M	10	22.9	13	1
2	71	F	10	0	18	3
2	30	M	10	34.1	3	2

Group 1 = abnormal TBUT, group 2 = normal TBUT; TBUT: tear film break-up time; OSDI: Ocular Surface Disease Index, normal: 0–12 points, mild 13–22, moderate 23–32, severe 33–100; Schirmer: Schirmer tear test; OSS: ocular surface staining, 0 = absent, 3 = severe.

## Data Availability

Data can be obtained from the corresponding author upon request.
